# Mitochondria in Myelinating Oligodendrocytes: Slow and Out of Breath?

**DOI:** 10.3390/metabo11060359

**Published:** 2021-06-05

**Authors:** Niklas Meyer, Johanne Egge Rinholm

**Affiliations:** 1 Division of Physiology, Institute of Basic Medical Sciences, University of Oslo, 0372 Oslo, Norway; 2 Department of Microbiology, Oslo University Hospital, 0373 Oslo, Norway

**Keywords:** oligodendrocyte, myelination, mitochondria, oligodendrocyte precursor cell (OPC), metabolism, ATP, glycolysis, oxidative phosphorylation, transcriptome, proteome

## Abstract

Myelin is a lipid-rich membrane that wraps around axons and facilitates rapid action potential propagation. In the brain, myelin is synthesized and maintained by oligodendrocytes. These cells have a high metabolic demand that requires mitochondrial ATP production during the process of myelination, but they rely less on mitochondrial respiration after myelination is complete. Mitochondria change in morphology and distribution during oligodendrocyte development. Furthermore, the morphology and dynamic properties of mitochondria in mature oligodendrocytes seem different from any other brain cell. Here, we first give a brief introduction to oligodendrocyte biology and function. We then review the current knowledge on oligodendrocyte metabolism and discuss how the available data on mitochondrial morphology and mobility as well as transcriptome and proteome studies can shed light on the metabolic properties of oligodendrocytes.

## 1. Oligodendrocytes—The Myelin Forming Glia Cells of the Brain

Publicized by Virchow in 1856 and for a long time after, glia cells have been thought of as solely glue that is holding the neuronal tissue together (glia [Greek] = glue, neuroglia = neuronal glue). A shift in the early 20th century changed how we view glia cells today, from passive form-givers to active contributors to the brain and its proper function [1,2]. Glia cells can be separated between central and peripheral nervous system (CNS and PNS). The different kind of CNS glia cells include radial glia cells, ependymal cells, microglia, astrocytes, oligodendrocyte precursor cells (OPCs, also called NG2-glia), and oligodendrocytes. Here, we briefly present the latter three groups of CNS glia. Astrocytes are the most abundant glial cell group with a myriad of interactions. Via their processes, they interact with all kinds of CNS cell types while providing metabolic support to neurons, recycling of neurotransmitters, spatial potassium buffering after neuronal activity, or help in the formation of the blood–brain barrier. In general, it can be said that astrocytes drive CNS development and modulate neuronal activity [3]. OPCs are the most proliferative cells in the CNS. The majority of OPCs differentiate into oligodendrocytes, although they are also able to form astrocytes and neurons [4]. After a pathological injury to the brain, they are essential to replenish the pool of functional cells, especially in human white matter [5,6]. Finally, mature oligodendrocytes are the myelin forming cells of the CNS. The myelin sheath is a lipid-rich membrane that extends from the oligodendrocyte processes and creates a dense wrap around axons, which allows for saltatory signal transduction of neuronal action potentials. A single oligodendrocyte is not only able to ensheath a single axon, but can rather form multiple axonal segments on different axons. Oligodendrocytes are much more abundant in the human than the murine brain, where they have been studied more extensively. This is illustrated by the large difference in grey- to white matter ratio in humans versus mice: the human brain volume comprises about 45% of white matter whereas the mouse brain shows only 10% [7,8,9]. It has been shown that when myelinating oligodendrocytes die, neuronal signaling fails and neurons are sure to follow [10]. Because of that, oligodendrocyte health and function is increasingly appreciated and studied.

## 2. Oligodendrocyte Maturation and Myelin Formation

The very first step of oligodendrocyte development marks the birth of a neural progenitor cell in the neural tube during embryogenesis which then differentiates to an OPC [11,12]. The OPCs further differentiate, through intermediate stages, into mature myelinating oligodendrocytes [13]. In the adult rat brain, OPCs comprise only 5–8% of the total glial cell mass with only a slightly smaller number in grey matter than in white matter [14]. Though, OPCs show region-specific behavior toward differentiation cues. For example, a subpopulation of white matter OPCs in organotypic slice cultures showed a stronger proliferative response and maturity toward platelet derived growth factor A (PDGF-A) stimulation than grey matter OPCs [15,16]. Additionally, in vivo experiments showed that more white matter OPCs develop to myelinating oligodendrocytes than grey matter OPCs, which remain NG2^+^ progenitors [17]. In the adult CNS, oligodendrocyte differentiation from OPCs is slowed down but never stopped [18] due to their role as a pool for new oligodendrocytes. The process of oligodendrocyte myelination is a complex and highly regulated sequence of events, which can be conceptualized into the following steps: migration and proliferation of OPCs, recognition of axon-glia signaling to find target axons, differentiation of OPCs into myelinating oligodendrocytes, membrane outgrowth to loosely wrap the axon, transfer of membrane components and proteins, and finally myelin compaction and node formation [19,20,21,22]. It could be shown that oligodendrocytes extend and retract their processes multiple times before deciding on the final position on an axon [20]. In addition, oligodendrocyte processes sense neighboring cells to ensure proper spacing of each myelin segment with evenly spaced nodes [21].

**Figure 1:**
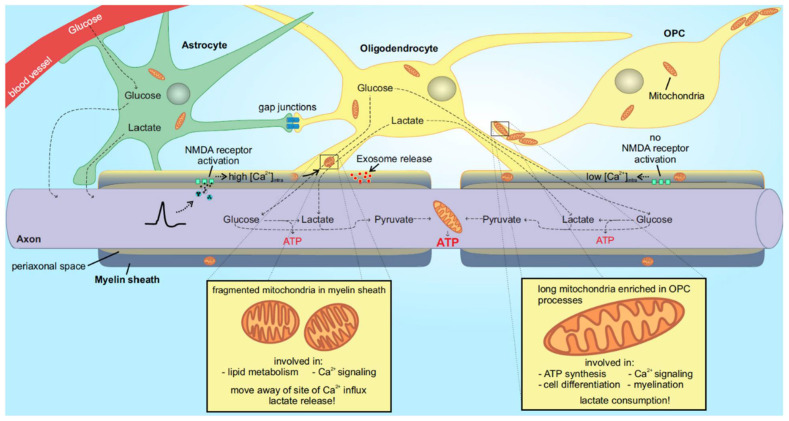
Metabolic fluxes between glia and axons and the proposed model of different functional states of mitochondria in oligodendrocytes during development. Myelinating oligodendrocytes metabolically support their isolated axonal segments in different ways via the direct transport of glucose and lactate and via the release of exosomes secreted from the myelin sheath. This support is only possible due to gap junction-coupled astrocytes that spread metabolites in the network. Oligodendrocytes sense axonal activity via NMDA receptors on the myelin sheath. This increases the intracellular Ca^2+^ concentration in the sheath, which causes lactate release. Since the small mitochondria in the myelin sheath move when Ca^2+^ is elevated, their movement away from the site of influx will facilitate release of lactate instead of lactate consumption. Proposed functions of myelin mitochondria include lipid metabolism and Ca^2+^ signaling. In contrast, mitochondria located in OPCs and immature oligodendrocytes show elongation and enrichment in the processes. These mitochondria have high oxidative phosphorylation (thereby consuming lactate) and are involved in Ca^2+^ signaling, cell differentiation, and initiation of myelination. See the main text for more details and references.

Myelination is regulated by multiple cues. For example, only axons with a large diameter are myelinated and myelination itself increases the axonal diameter not only by the deposition of myelin sheaths, but by the accumulation of neurofilament and its phosphorylation [23,24]. Myelin sheath formation is also regulated by Ca^2+^ activity in oligodendrocytes [25] as well as neuronal activity, which has also been shown to regulate OPC proliferation and differentiation [26,27,28]. Once formed, the myelin sheath provides the ability for rapid saltatory signal transduction as well as metabolic support to its underlying axons [29]. Metabolites and signaling molecules can pass from the oligodendrocyte soma through cytoplasm-rich myelinic channels in the compacted areas of myelin to the innermost tips of the oligodendrocyte processes enwrapping axons [22] (Figure 1). Furthermore, supporting and maintaining healthy myelin is dependent on the proper supply of metabolites. How, especially, oligodendrocytes are using this supply shall be examined in the next section.

## 3. Developing Oligodendrocytes Have Active Mitochondrial Respiration

The brain is by far the most energy demanding organ in the body: it is about 2% of the body weight, but consumes approximately 20% of the produced energy at rest [10,30]. Most of this energy enters the brain in the form of glucose, which crosses the blood–brain barrier and enters the brain cells via glucose transporters (GLUTs). Other metabolites such as lactate and ketone bodies can also be taken up from the blood stream and consumed by the brain. In addition to the uptake of metabolites from the blood, there is a transfer of metabolites between the different cell types in the brain. This has been studied thoroughly in neurons and astrocytes: while neurons have a high activity of oxidative phosphorylation (OXPHOS), astrocytes are shown to be highly glycolytic. It is therefore generally accepted that astrocytes release lactate (produced from glycolysis) that can be taken up by neurons and used for OXPHOS (Figure 1). This is called the astrocyte-to-neuron-lactate-shuttle and it has been shown to be crucial for neuronal function during high action potential firing or after an ischemic event [31,32].

How do oligodendrocytes fit into this scheme? The energetic demand of oligodendrocytes for the process of myelination is enormous. It has been estimated that the generation of 1 gram of myelin demands 3.3 × 10^23^ ATP molecules. The metabolites to generate this ATP have to be supplied from the bloodstream or from glycogen reserves of gap junction-coupled astrocytes [33]. Too little energy will inhibit myelination, as hypoglycemic conditions lead to a loss in OPC migration and differentiation or even overall decrease in oligodendroglial lineage cells and axonal degeneration [34,35]. The high energetic cost of myelin synthesis is paid off because the myelin greatly reduces the energetic demand of axons and speeds up signal propagation [36]. Energy is largely supplied in the form of glucose, but myelinating oligodendrocytes also use lactate for ATP production and for carbon skeletons to synthesize myelin lipids [37,38]. Furthermore, lactate has been shown to promote cell cycling and OPC differentiation [39], and it can support myelination during hypoglycemia [35]. Thus, developing oligodendrocytes have a high mitochondrial metabolism and OXPHOS rate before and during myelination. In line with this, OPCs and developing oligodendrocytes have a high density of long, tubular mitochondria [40], a mitochondrial morphology that is thought to support high OXPHOS [41]. Mitochondria in OPCs and immature oligodendrocytes are clustered close to the tips of the processes and display Ca^2+^ signals upon neuronal activity [42]. These mitochondria are thought to play a role in the differentiation and initiation of myelin formation and at the same time their location ensures local ATP production at the site of myelin induction.

## 4. Oligodendrocyte Mitochondrial Load and OXPHOS Is Reduced after Myelination

In adult mice, the grey matter is more energetically demanding than white matter, suggesting that oligodendrocytes have a lower energy demand after myelination is completed [43]. While developing oligodendrocytes are enriched with long mitochondria that support respiration, mature oligodendrocytes have a lower density of mitochondria. These mitochondria are not only fewer, but also more fragmented, at least in the processes [40,44]. In cultured OPCs, mitophagy was increased during OPC differentiation and inhibition of mitophagy arrested the differentiation [40]. Thus, reducing the number and size of mitochondria could be a necessary part of oligodendrocyte development. When compared with neurons and astrocytes, mitochondria in the processes of mature oligodendrocytes are about half the length and the mitochondrial density is half of that in neurons (Table 1, [44,45]). This shows that mature oligodendrocytes have a mitochondrial distribution and morphology that is distinct from any other brain cell studied. Moreover, the change in mitochondrial morphology and density between OPCs and myelinating oligodendrocytes suggests a shift towards less OXPHOS in mature cells, since a lower mitochondrial load and fragmented morphology is consistent with lower respiratory capacity. A metabolic switch during differentiation is also seen in other cell types and has been suggested as a driver of differentiation [46,47]. However, the oligodendrocyte metabolic switch appears to be in the opposite direction of that in neurons: neural stem cells have a high glycolytic activity, but switch to more OXPHOS in the transition to intermediate precursor cells [46,47]. Several studies support the idea that mature oligodendrocytes have low OXPHOS and higher glycolytic rate. White matter in rats was shown to exhibit a more glycolytic than oxidative metabolism, which could lead to an excess production of pyruvate and lactate [43]. Moreover, Fünfschilling et al. [48] knocked out OXPHOS specifically in oligodendrocytes. When this was done during oligodendrocyte development, it led to severe dysmyelination. However, when OXPHOS was knocked out after myelination was completed, there were no visible effects on myelin or axonal function. The authors concluded that oligodendrocytes have the ability to switch to a complete glycolytic state. Instead of consuming lactate, the mature oligodendrocytes release the lactate via the myelin sheath, which can be used by the underlying axons. The supply of metabolites via myelin to the axon seems like an elegant solution since the myelin sheaths also act as barriers that restrict the axons’ access to extracellularly supplied metabolites. In addition, long axons require a steady supply of metabolites from external sources to maintain signal transduction [29].

Lactate is transported across cell membranes via monocarboxylate transporters (MCTs). MCT1, MCT2, and MCT4 are expressed in the brain [56], where oligodendrocytes and their myelin sheaths exhibit MCT1 expression [35,48,57]. The importance of MCT1 for metabolic support is shown by a depletion experiment, in which MCT1 was depleted only in oligodendrocytes [57]. This led to extensive neuronal death, which could only be rescued by the supplementation of extracellular lactate [57]. A more recent study showed that loss of MCT1 early in development and myelination could be well tolerated, but led to late-onset hypomyelination and axonal degeneration in adult mice [58]. Lactate release from the myelin sheath is increased upon neuronal activity. This involves activation of N-methyl D-aspartate (NMDA) receptors that are present in the myelin sheaths of oligodendrocytes. When activated, Ca^2+^ influx through the NMDA receptors leads to increased expression of glucose transporter GLUT1, which is then transported into the cell membrane of oligodendrocytes to promote the influx of glucose. The glucose influx is followed by elevated glycolysis and lactate release to further support axonal function [59] (Figure 1). Recently, it could be shown in acute mouse brain slices that also glucose by itself rather than lactate seems to be enough to support axonal function after a period of aglycemia: oligodendrocytes in the corpus callosum released glucose and not lactate to the axons [60]. The picture is getting more nuanced by looking at another recent paper. Here, extracellular vesicles with properties of exosomes were released by oligodendrocytes and taken up by axons in order to metabolically support them and promote axonal transport in nutrient-deprived neurons [61] (Figure 1). However, precisely which metabolites or small molecules are getting transported via this route remains elusive.

Another important factor for metabolic support of neurons by glia cells is their interplay. In addition to the gap junction coupling between individual oligodendrocytes, astrocytes are coupled to oligodendrocytes via gap junctions. This enables the flux of metabolites and other substances directly between these cell types without the need for passage via the extracellular space [62]. The so called panglial coupling between oligodendrocytes and astrocytes is important for proper metabolic supply of axons during high energy demands. Astrocytes have been shown to support neuronal bodies and axons at nodes of Ranvier whereas oligodendrocytes do so via their myelin sheaths. However, intact gap junctional coupling is also a prerequisite for myelin maintenance and axonal function [60,63,64]. The necessity of functionally coupled astrocytes for myelin formation and maintenance could be shown in a study in which ablation led to myelin degradation. This in turn could be prevented by the addition of NMDA receptor antagonists, possibly hinting at a calcium buffering effect by astrocytes [65].

## 5. Mitochondria in the Myelin Sheath

It was long thought that the CNS myelin sheath was devoid of mitochondria, because there were no reports demonstrating their presence. In the peripheral nervous system, however, mitochondria have been detected in the cytoplasmic inner and outer tongues of the myelin sheath [66]. In 2016, we were able to demonstrate the presence of mitochondria in CNS myelin sheaths in organotypic brain slices and in vivo in mice by expressing mitochondrially targeted fluorescent protein specifically in oligodendrocytes [44]. The presence of mitochondria in CNS myelin sheaths has since been confirmed by two other studies [49,67]. Mitochondria are located mainly in cytoplasmic channels lining the compact myelin and in the paranodes. The myelin sheath mitochondria are smaller and the density less than a third of that in primary processes (Table 1). One reason for oligodendrocytes to stay small and thereby keep a low OXPHOS could be reactive oxygen species (ROS) that accumulate during mitochondrial respiration. OPCs and oligodendrocytes are particularly vulnerable to ROS due to their low concentrations of antioxidant enzymes like glutathione peroxidase and catalase [68,69]. Unlike oligodendrocytes, OPCs show morphological anomalies upon metabolic injury due to their high mitochondrial demands, demonstrating their susceptibility to stress [70].

Interestingly, while inhibition of mitochondrial fission by blocking the Dynamin-related protein 1 (Drp1) with mdivi-1 protects neurons against excitotoxicity, mdivi caused mitochondrial depolarization in oligodendrocytes and sensitized them to oxidative stress [71]. It was also demonstrated that oligodendrocytes shift under stress conditions to a more glycolytic metabolism in favor of survival rather than myelin maintenance [72]. Taken together, these findings suggest that the oligodendrocyte mitochondria, and in particular those located in the myelin sheath, have a relatively low OXPHOS activity. Therefore the main function of myelin mitochondria must be something other than ATP production. In adipocytes, induction of small, round mitochondria can reduce lipolysis by reducing the contact surface between the mitochondria and lipid droplets [73]. A similar reason could be true in oligodendrocyte myelin where too much mitochondrial beta-oxidation would break down the lipid-rich myelin. Thus, small fragmented mitochondria could prevent the breakdown of the myelin sheath. Instead, these mitochondria may support lipogenesis by providing citrate via the tricarboxylic (TCA) cycle. Another function of mitochondria is regulation of calcium homeostasis. Battefeld et al. [67] recently showed that myelin mitochondria contribute to calcium transients in the myelin sheath. In fact, the mitochondria in the myelin sheath, and not neuronal action potentials, were generating these calcium transients (although neuronal activity can induce Ca^2+^ signals in the myelin sheath [74]). The mitochondria-induced calcium signals were most frequent during myelination and remyelination and might be integral in the myelination process [67]. Thus, the small round mitochondria of the myelin sheath may play an important role in myelin Ca^2+^ dynamics as well as supporting maintenance of the lipid-rich myelin membrane.

## 6. Mitochondrial Mobility in Oligodendrocytes

Mitochondria are highly dynamic organelles. They are transported around the cell along cytoskeletal tracts and can alter their size by undergoing fission and fusion. These mitochondrial dynamics serve several functions: The balance of fission and fusion regulates the length of mitochondria and is influenced by stress and availability of nutrients [55]. Furthermore, the movement and fragmentation are important for the removal of damaged mitochondria, which are then cleared by mitophagy. Finally, the mitochondrial movement is thought to serve the purpose of redistributing mitochondria to parts of the cell that are in need of energy. For instance, in neurons, mitochondria are recruited to active synapses. The movement and stopping of mitochondria in neurons seem to serve the purpose of increasing OXPHOS (and thereby ATP production) at active sites [75].

The role that mitochondrial dynamics play in oligodendrocytes is not well understood. By performing live imaging in organotypic mouse brain slices, we demonstrated that oligodendrocyte mitochondria move along primary processes and are able to enter and move within the myelin sheath [44]. The oligodendrocyte mitochondria showed a lower mobility compared with neurons and astrocytes: a smaller fraction of the oligodendrocyte mitochondria were moving and they moved slower (Table 1, [44,45,50]). Nakamura et al. [49] reported a mitochondrial velocity in primary oligodendrocyte cultures that was about ten times higher than our findings. The discrepancy could be due to differences between primary cultures and slices: also in neurons, studies from primary cultures tend to report higher velocities than those from tissue cultures and in vivo imaging ([44,45,50,52] and Table 1). It is likely that the mitochondrial velocity is reduced by different signals that make the mitochondria slow down or stop. For example, Nakamura et al. [49] showed that oligodendrocyte mitochondria slowed down in response to netrin-1 signaling. In neurons, mitochondria are stopped by glutamate signaling, as discussed in more detail below. It is not known why oligodendrocyte mitochondria have lower mobility than mitochondria in other studied cell types. We speculate that higher mitochondrial mobility is necessary in cells with long processes (i.e., in neurons) where the OXPHOS activity is high, not only to be able to distribute mitochondria to areas in need of energy, but also to enable fission and mitophagy of damaged mitochondria, which would presumably occur more in cells with high OXPHOS due to their increased ROS production [76]. Thus, the shorter length of cell processes and the lower OXPHOS may explain the low mobility of oligodendrocyte mitochondria. Of note, astrocytes, which are closer in size to oligodendrocytes and are considered highly glycolytic, but do still have a significant level of respiration [77], have values that lie between neurons and oligodendrocytes when it comes to mitochondrial mobility (Table 1). A likely consequence of the low density and mobility of oligodendrocyte mitochondria is less fusion and thereby less exchange in mitochondrial content. This could, in turn, lead to mitochondrial heterogeneity, as was shown for astrocytes [78]. Such heterogeneity in oligodendrocytes could, for instance, involve higher rates of OXPHOS in mitochondria located in the cell soma compared with myelin mitochondria.

In neurons, mitochondria move along microtubules by linking to kinesin or dynein motor proteins. Mitochondrial coupling to the motors is aided by several adaptor proteins such as Miro1 and Milton [55]. In addition, mitochondria are thought to have short-range movement along actin filaments, facilitated by myosin motors. Cells also have a stationary pool of mitochondria, which are tethered to the cytoskeletal filaments via specific anchoring proteins [55]. Studies from neurons and astrocytes demonstrate that mitochondria are stopped at sites of increased Ca^2+^ influx during glutamate signaling [45,50]. In neurons, the Ca^2+^ entering through NMDA receptors stops mitochondria at active synapses. This mechanism depends on Ca^2+^ binding to Miro1 [50], thereby detaching mitochondria to the kinesin motor. Mitochondria in astrocytes were stopped by Ca^2+^ influx through the Na^+^/Ca^2+^ exchanger (in reverse mode) during high neuronal activity [45], suggesting a similar mechanism to that in neurons. Surprisingly, mitochondria in oligodendrocytes were not stopped by glutamate. Instead, glutamate increased mitochondrial movement by 76% [44]. This appeared to be Ca^2+^ dependent as removal of Ca^2+^ from the extracellular medium completely abolished mitochondrial movement. Thus, it seems that mitochondrial movement in oligodendrocytes is not reduced, but rather increased by Ca^2+^ influx. This implies a completely different mechanism for mitochondrial movement and stopping in oligodendrocytes compared with other brain cells. ATP availability has been suggested as another regulator of mitochondrial movement, but the mechanisms for this are unclear [79]. The increased glucose uptake of oligodendrocytes after NMDA receptor activation would lead to increased ATP availability (produced by glycolysis) [59]. Moreover, if elevated Ca^2+^ leads to increased mitochondrial movement in oligodendrocytes, this would cause mitochondria to move away from the NMDA-mediated Ca^2+^ influx sites, thus ensuring that lactate is not metabolized and can instead be released (Figure 1). Since mitochondrial Ca^2+^ uptake stimulates ATP production (via activation of mitochondrial dehydrogenases and ATP synthase, [80,81]), moving away from Ca^2+^ influx sites would prevent Ca^2+^-induced mitochondrial ATP production. From a functional point of view, if oligodendrocyte mitochondria mainly serve purposes other than ATP production, then it makes sense that they should not be stopped at active sites in the same way as in neurons and astrocytes. It should be mentioned that the netrin-1-dependent reduction of mitochondrial movement [49] may contradict these findings since extracellular netrin-1 leads to increased intracellular Ca^2+^, at least in neurons [82]. Therefore the effects of intracellular Ca^2+^ should be studied further by combined imaging of intracellular Ca^2+^ and mitochondria in oligodendrocytes.

## 7. What Can We Learn about Oligodendrocyte Metabolism from Transcriptome and Proteome Data

In recent years, several groups have published transcriptome or proteome data in which they compared gene expression between oligodendrocytes at different developmental stages and/or between oligodendrocytes and other brain cells. If mature oligodendrocytes are highly glycolytic with low OXPHOS, this should be reflected in their transcriptome and proteome profile. Indeed, the transcriptome profile has been used to argue for the high glycolytic activity of astrocytes and the astrocyte-to-neuron lactate shuttle [83]. Here, we compared transcriptome and proteome data of four key metabolic enzymes from four different studies [83,84,85]. We present figures made from expression tables available from two of the papers, namely Zhang et al. [83] and Sharma et al. [84] for transcriptome and proteome analysis, respectively. These papers were chosen for figures because they contain data from all the relevant cell types and because the data expression tables were available.

**Figure 2:**
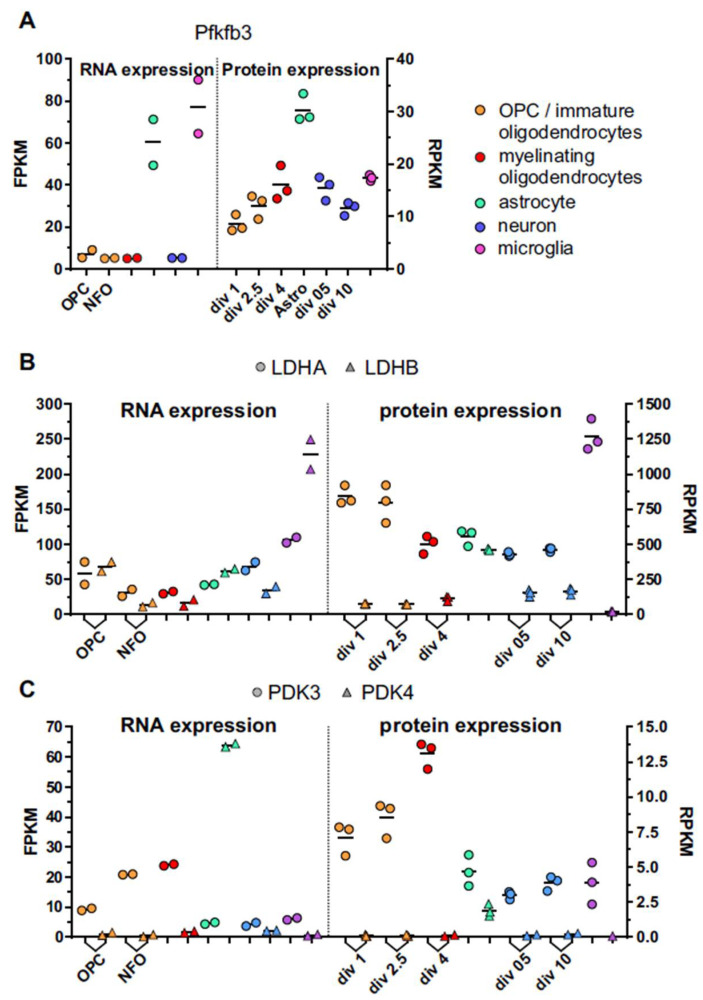
RNA and protein expression of regulatory enzymes involved in glucose and pyruvate metabolism. Transcriptome and proteome data are from Zhang et al. [83] and Sharma et al. [84], respectively. Different cell types are indicated by different colors as explained in the top right panel. The study by Sharma et al. used isolated cells that were cultured and analyzed at different days in vitro (DIV), as indicated. (**A**) Expression of Pfkfb3, a major positive regulator of glycolysis. (**B**) Expression of the two lactate dehydrogenase isozymes LDHA and LDHB. (**C**) Expression of PDK3 and PDK4, which indirectly act as positive regulators of glycolysis by inhibiting pyruvate metabolism in mitochondria. See main text for details.

Pfk1 (6-phosphofructo-1-kinase) is a master regulator of glycolysis. It is activated by the enzyme fructose-2,6-bisphosphate, which is generated by Pfkfb3 (6-phosphofructo-2-kinase/fructose-2, 6-bisphosphatase-3). Therefore, the level of Pfkfb3 is used as a measure of glycolytic activity, and is highly expressed in glycolytic cancer cells [86]. In line with this, neurons are unable to upregulate glycolysis due to low Pfkfb3, whereas glycolytic astrocytes have high Pfkfb3 [83,87]. Thus, if mature oligodendrocytes are highly glycolytic, a high level of Pfkfb3 would be expected. Surprisingly, the mRNA and protein levels of Pfkfb3 in oligodendrocytes appears to be similar to that in neurons, and significantly lower than that in astrocytes [83,84,85] (Figure 2A). Whether there is a change in expression of Pfkfb3 during oligodendrocyte maturation is unclear as there is a discrepancy between the different studies. While the proteome study by Sharma et al. [84] and the transcriptome study by Cahoy et al. [85] suggest an increase in Pfkfb3 protein during oligodendrocyte development, this was not seen in the study by Zhang et al. ([83], Figure 2A). In Marques et al. [88], Pfkfb3 is elevated by a factor of 3 in immature myelin-forming oligodendrocytes compared with OPCs, but then goes down again in mature oligodendrocytes. The discrepancy between the different studies could be due to differences in cell sorting methods or in situ isolation versus culture. Of note, the levels of Pfkfb3 in microglia were as high at those in astrocytes (Figure 2A). This suggests that microglia are highly glycolytic, as recently reported [89]. However, microglia may have a high metabolic flexibility, and change toward more glycolysis, specifically when activated [90].

Another critical regulated point of glycolysis is the final step, in which phosphoenol pyruvate is converted to pyruvate through the enzyme pyruvate kinase (PK). PK is found in two splice variants, Pkm1 and Pkm2. In contrast to Pkm1, Pkm2 contains an inducible nuclear translocation signal that allows a cell to regulate the amount of glycolytic flux in response to the local energy state. The study by Zhang et al. [83] showed that while neurons only have Pkm1, locking them in a steady state of glycolysis, all glia cells including oligodendrocytes express Pkm2.

The pyruvate produced by glycolysis is either transported into mitochondria for further metabolism or converted to lactate by lactate dehydrogenase (LDH). Conversely, LDH can convert imported lactate into pyruvate. LDH is a family of enzymes including five isoforms, each consisting of tetramers of M and H subunits. The M (muscle variant) and H (heart variant) subunits are encoded by the LDHA and the LDHB genes, respectively. The differential expression of LDHA and LDHB has been proposed to reflect glycolytic activity with LDHA preferentially facilitating pyruvate to lactate conversion and being expressed in tissue with high glycolytic activity [91], although some studies have stated the opposite [83]. There is inconsistency between the different transcriptome and proteome in oligodendrocyte expression of LDH variants, with the only common trend being that LDHA is reduced during oligodendrocyte maturation (Figure 2B, [83,84,85,88]). In any case, it is unclear whether the LDHA/LDHB ratio in itself can predict whether a cell type is mainly glycolytic as astrocytes predominantly express the LDHB variant (at least on RNA level) and neurons predominantly express LDHA ([83,85,92] and Figure 2B).

When pyruvate enters the mitochondria, it is converted to acetyl-CoA and CO_2_. This reaction is catalyzed by the pyruvate dehydrogenase (PDH) complex. Since PDH provides the primary link between glycolysis and the TCA cycle, it is one of the major enzymes responsible for the regulation of glucose metabolism. The activity of PDH is regulated by pyruvate dehydrogenase kinase (PDK), which inactivates PDH by phosphorylation. PDK exists in four isoforms, PDK1–4, all of which have somewhat different phosphorylation activity. While all isoforms seem present in the brain, PDK3 has the highest expression. Importantly, PDK4 might be particularly important in glycolytic cells as it is the only isoform that has a basal activity [93]. In agreement with this, astrocytes have particularly high PDK4 activity. In comparison, oligodendrocytes have little, if any, PDK4, but instead they have a high expression of PDK3 compared with the other cell types (Figure 2C). Since PDK3 does not have basal activity, these data may point to a high potential for inducing glycolytic activity in oligodendrocytes rather than a generally high glycolysis.

In sum, the different transcriptome and proteome studies usually show a similar pattern of expression, with some exceptions. Most studies show that astrocytes, which are known for a high glycolytic activity and lactate release, have an expression pattern for metabolic enzymes that differs from that of neurons and oligodendrocytes. Oligodendrocytes have an expression pattern that more closely resembles that of neurons, perhaps with the exception of LDHA and PDK3. This could mean that oligodendrocytes have a lower glycolytic rate than astrocytes, but with a large potential to increase the glycolytic rate when needed.

## 8. Conclusions

Mitochondria have many important functions throughout the life of oligodendrocytes. During development, oligodendrocytes have a large network of tubular mitochondria that are important for ATP production and Ca^2+^ signaling to stimulate differentiation and growth of the myelin sheath. After myelination is complete, mature oligodendrocytes have fewer and more fragmented mitochondria. This change in mitochondria is linked to a shift toward more glycolysis and less OXPHOS, and mature oligodendrocytes release lactate from the myelin sheath to underlying neurons. Mitochondria are also present within the myelin sheath, but their low density and small size indicate they are not important for OXPHOS. Though more research is needed to unravel the functions of these mitochondria, we propose that they are specialized for lipid metabolism and Ca^2+^ homeostasis. While oligodendrocyte mitochondria have low mobility compared with other brain cells, their movement increases upon Ca^2+^ entry through NMDA receptors. This suggests an entirely different mechanism for the regulation of mitochondrial movement than in other brain cells and would ensure mitochondria are removed from the myelin sheath when active neurons signal to receive lactate. Finally, expression of metabolic enzymes in oligodendrocytes resembles that of neurons more than astrocytes and microglia. This suggests that oligodendrocytes do not have as high basal glycolytic activity as astrocytes (and presumably microglia), but that they have the potential for inducing high glycolysis when needed.

## Figures and Tables

**Table 1:** Density, length, and mobility of mitochondria in different cell types and compartments of the nervous system.

Cell Type/Compartment.	Density 100 µm^−1^	Length µm	Velocity µm/s	% Moving (15/20 min)	References
Oligodendrocyte primary processes	8.7	1.2 2.3 ^1^	0.07 0.8–1 ^1^	12 (20 min)	[44,49]
Oligodendrocyte myelin sheaths	2.4	0.8	0.08	8 (20 min)	[44]
Astrocytes	-	2.5–3	0.15–0.2	20 (15 min)	[45]
Neuronal dendrites (CNS)	14^1^	2.5–3 2.2 ^1^	0.3 0.9 ^1^	40/50 (15/20 min)	[44,45,50,51]
Neuronal axons (CNS)	13–14 ^1^	1.4 ^1^	0.5–0.6 ^1^ 0.4–0.7 ^2^	-	[51,52] [53]
Schwann cell myelin sheaths	-	1–3 ^2^	0.14 ^2^	-	[54]

^1^ From primary cultures. ^2^ From in vivo measurements. All other values are from organotypic slice cultures. There is a lot of in vivo data published on mitochondrial movement in axons. These data were not included here due to differences in how movement was reported, but overall, in vivo data show somewhat lower mitochondrial mobility than in cell culture (for an overview, see [55]). We have not been able to find similar data for microglia. These cells are therefore not included in the table.
